# Application of a Telephone Program for Informal Caregivers of Patients with Bipolar Disease

**DOI:** 10.3390/jcm14228173

**Published:** 2025-11-18

**Authors:** Catarina Inês Costa Afonso, Ana Spínola Madeira, Alcinda Reis, João Gomes

**Affiliations:** 1 Integrated Member of RISE-Health, School of Health Sciences of Santarém, Polytechnic Institute of Santarém, Quinta do Mergulhão Srª da Guia, 2005-075 Santarém, Portugal; ana.madeira@essaude.ipsantarem.pt (A.S.M.); alcinda.reis@essaude.ipsantarem.pt (A.R.); 2 Local Health Unit of the Leiria Region, 2400-441 Leiria, Portugal; joaomfg@gmail.com

**Keywords:** bipolar disease, informal caregiver, psychoeducation, telephone support, mental health

## Abstract

**Background/Objectives:** Family caregivers of individuals with bipolar disorder (BD) experience substantial burden, yet scalable caregiver-focused supports are scarce. This pilot study tested a nurse-led telephone program to evaluate feasibility and acceptability and to explore perceived impacts on caregiver burden, coping, and well-being. **Methods:** A descriptive pilot case study was conducted in an adult psychiatric inpatient unit in Portugal. Six informal caregivers of inpatients with BD completed a structured six-call protocol over approximately 6–8 weeks. **Results:** Overall, 6 caregivers completed the full cycle, totaling 36 sessions (6 assessment, 18 psychoeducational, 6 psychosocial, and 6 evaluation sessions). Thematic analysis identified four recurrent themes: (1) embracing the caregiver role—recognizing personal needs and legitimizing help-seeking; (2) patience and understanding—adopting emotion-regulation strategies; (3) self-reflection on personal strengths—increased self-efficacy and acknowledgment of persistence, empathy, and resilience; and (4) fostering hope and resilience—expressing future-oriented goals consolidated in a personalized “hope kit.” **Conclusions:** A brief, protocolized, nurse-delivered telephone program for caregivers of inpatients with BD was both feasible and acceptable, producing meaningful qualitative benefits consistent with the aims of psychoeducation (knowledge acquisition, coping, and emotional regulation). Findings support the use of telephone support as a pragmatic complement to standard BD care and justify larger controlled studies to quantify effects on caregiver burden, mood, and resilience, and to compare telephone, in-person, and blended delivery models.

## 1. Introduction

Bipolar disorder (BD) is a severe, chronic mental illness characterized by dramatic swings between manic and depressive episodes [1]. These extreme mood fluctuations not only disrupt the lives of patients but also impose significant challenges on their families. In particular, family members who act as caregivers often experience considerable emotional and physical strain in coping with the unpredictable and sometimes volatile nature of BD symptoms [2]. BD is relatively prevalent—affecting roughly 1–3% of the population—and it ranks among the leading causes of disability worldwide [1]. Its chronic, recurrent course can have a “ruinous effect” on caregivers’ quality of life, impacting their mental health, work productivity, and social relationships [3]. Without adequate assistance, many caregivers face burnout, a state of physical and emotional exhaustion that not only harms their own health but can also impair their caregiving capacity [3,4]. Family caregivers of BD patients often experience high subjective distress (e.g., stress, guilt) and objective burdens (e.g., financial costs, time demands). Many describe feelings of hopelessness or being “at the end of [their] rope” due to relentless caregiving demands. Unfortunately, caregivers’ physical, emotional, and financial well-being is frequently overlooked in traditional patient-centered care, which can exacerbate burnout and undermine both caregiver and patient outcomes. It is thus increasingly recognized that caregiver support should be integrated into BD treatment to improve outcomes for the whole family [3,4,5,6]. Multiple studies have shown that those caring for individuals with mental illness tend to experience greater burden than caregivers of patients with other chronic medical conditions [5,7,8]. For example, previous studies report moderate caregiver burden and low resilience among family members of BD-I patients, and they specifically recommend psychoeducational interventions to alleviate this burden [5]. In another study, a cross-sectional survey of psychiatric caregivers, approximately 60% reported a moderate level of burden, and about 40% reported high burden, indicating that the majority of families shoulder significant stress in the caregiving role [7]. Such caregiver burden is associated with a lower quality of life and elevated rates of depression and anxiety among caregivers [3,8]. Indeed, depressive symptoms are common in this population—many caregivers meet criteria for clinical depression—which can undermine caregivers’ ability to continue providing effective support [5,7,8]. In light of this, experts emphasize that caregivers need robust social and professional support to prevent burnout and maintain their well-being [9]. However, families commonly report a lack of formal support and education in managing their relative’s mental illness, which further exacerbates the strain [6]. Stigma and social isolation may additionally compound the stress, as caregivers of psychiatric patients often have fewer outlets for respite or understanding in their communities [7]. These challenges highlight a clear gap in standard mental health services: the caregivers of patients with BD require attention and resources in their own right. Supporting caregivers is not only a compassionate or altruistic concern, but also pragmatic, because a well-supported caregiver is better equipped to contribute to the patient’s stability and recovery [6].

Traditional family-focused therapies and psychoeducation programs in BD have typically involved both the patient and their relatives, aiming to reduce relapse rates through improved illness management at home. For instance, adding family psychoeducation to standard pharmacotherapy has been shown to significantly delay mood episode recurrences in patients with BD [10,11]. In one landmark randomized trial, patients receiving a family-focused psychoeducational therapy (in conjunction with medication) had longer relapse-free periods and fewer mood episodes over one year compared to patients receiving medication alone [11]. Notably, caregiver-only interventions can also positively influence the course of the illness: a group-based psychoeducation program delivered exclusively to caregivers (with patients not attending) led to longer relapse-free intervals and a lower incidence of manic/hypomanic episodes in their bipolar relatives, relative to a control group with no caregiver intervention [12]. This suggests that empowering and educating caregivers can indirectly benefit patients—likely by improving medication adherence in the household, enhancing early detection of mood changes, and fostering a more stable, supportive home environment.

Importantly, caregiver-oriented interventions have also been found to benefit the caregivers themselves. In one study, caregivers who participated in a 12-week family psychoeducation program showed a significant reduction in subjective burden (their perceived stress and emotional distress) and improved knowledge about BD, compared to caregivers receiving no intervention [4]. Although objective burden (tangible caregiving duties and time demands) did not change in that short-term study, the alleviation of caregivers’ emotional strain is a meaningful outcome, since subjective burden is closely linked to caregiver depression and anxiety [4,8]. These findings align with other research indicating that psychoeducational and skills-training interventions can help caregivers reframe their situation, feel more competent in their caregiving tasks, and ultimately experience less stress [9]. Despite such evidence, specialized programs focusing on the caregiver’s own needs and mental health remain relatively scarce in routine practice. Many healthcare systems still focus predominantly on the patient, often leaving family members to learn “on the job” with minimal guidance or support [6]. This underscores a critical need to develop and implement accessible support programs for family caregivers of people with BD, in order to enhance their coping skills, resilience, and well-being alongside that of the patient [6]. In summary, there is a compelling rationale to integrate formal caregiver support into psychiatric care—not just for humanitarian reasons, but also because bolstering caregiver well-being can improve patient outcomes.

One promising avenue for delivering caregiver support is through telehealth and telephone-based interventions. Telephone support programs offer a flexible, accessible means to reach caregivers. Caregivers may have limited time, transportation, or local resources for in-person help. Moreover, remote interventions gained particular importance during the COVID-19 pandemic, when face-to-face services were restricted—highlighting that technology can bridge the gap and provide needed assistance to families in isolation [13]. Recent studies have shown that such approaches can be effective. For example, a randomized controlled trial in dementia care demonstrated that a telephone-based psychoeducational intervention (10 structured phone sessions over 12 weeks) led to substantial relief for caregivers: those who received scheduled phone counseling had significantly lower Zarit burden scores and reduced anxiety/depression symptoms post-intervention, relative to control caregivers who did not receive the calls [14]. These examples illustrate that telephone-delivered psychosocial support—whether via voice calls or combined with digital tools—can feasibly reduce caregiver stress and improve psychological outcomes. Telehealth interventions have the added advantage of convenience and scalability, allowing outreach to many caregivers without the barriers of travel or scheduling group meetings. For caregivers of people with psychiatric illnesses, who often juggle caregiving with other responsibilities and may have their own health constraints, the accessibility of telephone support is especially beneficial. It permits caregivers to receive psychoeducation (about topics like medication adherence, symptom management, and crisis planning) and psychotherapeutic support (such as counseling on coping strategies and stress management techniques) from home, at flexible times. By lowering the threshold to engage in support services, telephone programs can engage caregivers who might otherwise not seek help. Early evidence suggests that these remote interventions can improve caregivers’ confidence and reduce feelings of isolation—factors which are key to mitigating subjective burden and preventing caregiver burnout [13].

In light of the above considerations, a telephone-based support program was developed for family caregivers of individuals with BD admitted to a psychiatric inpatient unit. This nurse-led program was designed to provide structured support through regular phone calls, combining psychoeducational sessions (to enhance caregivers’ understanding of bipolar disorder and effective care strategies) with psychosocial counseling (to address emotional needs, stress management, and coping skills).

This study introduces an innovative, nurse-led telephone program specifically designed to support informal caregivers of individuals hospitalized for bipolar disorder. Unlike traditional family interventions that are clinic-based or involve multidisciplinary teams, this model centers on nurses as primary facilitators of structured, six-session psychoeducation and emotional support. Its brief, protocolized format and exclusive focus on caregivers during the inpatient phase represent a novel, pragmatic approach to bridging the gap between hospital care and community follow-up [9,10,11,12,13,14].

The present article reports on the implementation and impact of the telephone support program. The program’s success was evaluated primarily in terms of its impact on the caregivers themselves—namely, changes in perceived burden, attitudes, and coping abilities after participation. This focus on caregiver outcomes (rather than just program adherence or satisfaction) aligns with evaluation frameworks that emphasize outcomes over process when assessing healthcare interventions [6]. To capture these outcomes, narrative feedback from caregivers at the conclusion of the program was gathered, and recurring themes were analyzed using a qualitative, thematic approach. The objectives of this study were twofold: (1) to describe the development of a nurse-led telephone support intervention for family caregivers of bipolar disorder patients, and (2) to assess its impact on caregiver burden and well-being through the caregivers’ own reported experiences. It was hypothesized that caregivers engaging in the program would report reduced feelings of overload and enhanced coping and emotional resilience.

## 2. Materials and Methods

### 2.1. Study Design

This study followed a descriptive, exploratory mixed-method pilot case-study design aimed at evaluating the impact of a nurse-led caregiver support program. The program’s success was assessed in terms of its impact on caregiver outcomes (perceived burden, coping, and well-being) rather than just the implementation process [15,16]. The project was reviewed and approved by the institutional ethics committee of Hospital of Santarém, Portugal (June 2021).

Accordingly, the success of the telephone support program was assessed in terms of its impact on caregiver outcomes, as reported by the caregivers themselves. The evaluation criteria were defined by the program’s objectives, determining the degree of success in achieving those objectives based on predefined outcome standards.

This approach aligns with recommended evaluation frameworks that prioritize outcome (result) indicators over output or activity metrics when assessing healthcare interventions [15,16].

### 2.2. Setting and Participants

The study was conducted in the adult psychiatric inpatient unit of a District Hospital in Portugal. Inclusion criteria for caregivers were: being a primary family caregiver of a hospitalized BD patient, availability for telephone sessions, and providing informed consent. At baseline (prior to the intervention), caregivers completed the Scale Caregiver Burden (SCB), a Portuguese adaptation of the Zarit Burden Interview, to quantify their perceived burden. The SCB is a 21-item instrument measuring subjective caregiver strain with total scores ranging from 0 (no burden) to 88 (extreme burden).

The program was implemented over an 8-week period (from June to August 2021) during which all patients with BD admitted to the unit were screened for eligible family caregivers. A total of 34 patients were admitted in this period (mean age 48 years, mode 53). Of these, 21 patients had an identified family caregiver involved in their care, 6 of them with BD. 

### 2.3. Intervention Protocol

The program is a nurse-led telephone support developed to provide structured psychoeducational and emotional support to family caregivers of patients with BD. The intervention was delivered by a mental health nurse via regular phone calls, allowing flexibility and access for caregivers who might face barriers to in-person support. Each caregiver received a series of six phone sessions over the course of the patient’s hospitalization and immediate post-discharge period (typically spanning about 6–8 weeks). The content and structure of these sessions were standardized according to a written protocol, ensuring consistency across participants. In total, 36 calls were completed (6 per each of the 6 caregivers) in accordance with the program design. The sessions were scheduled at mutually convenient times, and each call lasted approximately 30–45 min.

The program combined psychoeducation about bipolar disorder with psychosocial counseling and skills training. The sequence of telephone sessions for each caregiver was as follows:

- Initial Assessment Call: A baseline session to establish rapport, assess the caregiver’s situation and needs, and create awareness of the caregiver role. During this call, the nurse collected background information and administered baseline measures (e.g., caregiver burden scale). Caregivers were encouraged to share their caregiving history and feelings, which often elicited emotional narratives. For example, many caregivers described a profound sense of responsibility and recognized the need for support or a “cane” (metaphorically) to help them continue caring. This initial reflection helped caregivers acknowledge their role and the importance of seeking help (“I realized today that I need a cane,” noted one participant, indicating recognition of needing support).
- Psychoeducational Sessions (3 calls): Three structured calls focused on providing information and guidance about BD management at home. These sessions covered topics such as understanding BD and its mood episodes, medication adherence, and the importance of continuing treatment after discharge, recognizing early warning signs of relapse, communication strategies to improve interaction with the patient, and crisis planning. The nurse delivered tailored education and answered questions, while also addressing caregivers’ misconceptions or fears. Interactive techniques were used to engage the caregiver–for instance, exploring the difference between emotions and feelings, and discussing how the patient’s behaviors can trigger emotional reactions. Caregivers learned coping strategies like the “turtle technique” or the “10 s rule” (simple calming exercises) to manage frustration, especially in situations such as when patients refuse medication or exhibit challenging behaviors. All caregivers reported these practical techniques to be useful in helping them regulate their emotions, leading to responses like: “It’s true, I end up losing patience, but counting to ten or withdrawing for a moment helps me not to explode.” These sessions aimed to increase caregivers’ patience, understanding, and skill in handling the unpredictable nature of BD, thereby reducing stress in the caregiver-patient relationship.
- Psychosocial Support Session (1 call): One telephone session was dedicated to therapeutic listening and counseling, allowing caregivers to express their feelings, challenges, and emotional distress. The nurse provided empathic support and guided the caregiver in reflecting on their own well-being and coping capacity. An important component was helping caregivers identify their personal strengths and resilience factors. Caregivers engaged in self-reflection exercises during this call–for example, each was prompted to acknowledge positive qualities in themselves (such as being persistent, compassionate, a good problem-solver, or friend). This exercise yielded moments of lightness and empowerment; as one caregiver remarked, “It’s so good to think about what I’m good at, to recognize my abilities.” Such discussions fostered a sense of self-efficacy and helped reframe caregivers’ self-image from feeling overwhelmed to also seeing themselves as resourceful and capable. Techniques for positive self-talk, optimism, and even the use of humor to cope were discussed to bolster the caregivers’ emotional resilience.
- Final Evaluation Call: The final session concluded the intervention and evaluated its perceived impact. In this call, the nurse and caregiver reviewed the journey since the first session, reflecting on any changes in the caregiver’s feelings of burden, attitude, or coping strategies. Caregivers were invited to provide narrative feedback about their overall experience with the program. Open-ended questions (e.g., “How do you feel this program has helped you?”) encouraged caregivers to articulate any perceived benefits or remaining challenges. A guided visualization exercise—using the metaphor of a “magic wand”—was employed to help caregivers express their hopes for the future. Caregivers imagined if they had a magic wand, what would they wish for themselves, their loved one, or their situation (common wishes included “hope,” “peace and patience,” “strength and courage,” “better health,” and “more time and understanding”). This exercise instilled a sense of hope and highlighted the caregivers’ aspirations, reinforcing the theme of resilience. Following this, the nurse summarized key takeaways and coping tools from all prior sessions to create a “hope kit” for the caregiver—essentially a personalized compilation of insights, strategies, and encouraging reminders that the caregiver could carry forward. In closing, caregivers were thanked for their participation and reminded of available ongoing support. The final session was also an opportunity to evaluate the program’s success criteria: reduced subjective burden, improved coping, and positive attitude changes as described by the caregivers in their own words.

Throughout all sessions, the nurse maintained a person-centered approach, fostering a safe and trusting environment for the caregivers to share openly. Any acute needs or issues that emerged were addressed or referred appropriately (for example, advising on seeking additional psychiatric consultation if a patient showed severe warning signs, or connecting the caregiver with social services if needed). The intervention was thus not only structured and educational but also flexible and responsive to individual caregiver circumstances.

### 2.4. Data Collection

Data were collected from participants at two main points: at enrollment (pre-intervention baseline) and at the end of the program (post-intervention). At baseline, a brief questionnaire was used to gather caregiver demographic information (age, gender) and caregiving context (relationship to patient, duration of caregiving in years, and estimated hours per day devoted to caregiving). The baseline Caregiver Burden Scale was implemented, and the score was recorded for each participant. These quantitative data were used to characterize the sample and confirm the high burden levels at the start.

During the final evaluation call, qualitative data were collected in the form of narrative feedback. Each caregiver was asked open-ended questions to elicit their personal experience with the program and any perceived changes in their well-being or caregiving outlook. Example prompts included: “Can you describe how you felt at the beginning of this program and how you feel now regarding your caregiving role?”; “What was the most helpful aspect of these calls for you?”; “Did you notice any changes in your stress levels or how you cope with challenges?”; and “What are your hopes moving forward after completing this program?”. Caregivers’ responses were encouraged to be as detailed as possible. The nurse conducting the calls took detailed notes (and, with permission, some calls were audio-recorded to ensure accuracy of the feedback). These narrative responses captured the subjective impact of the program on the caregivers—including emotional reactions, changes in attitude, and any new strategies they adopted. After the program’s completion, the narrative feedback from all 6 caregivers was compiled for analysis.

In addition to the narrative data, the program’s operational data were documented: the number of sessions completed per caregiver, session attendance/adherence, and any drop-outs or missed calls. In this pilot, adherence was high—all 6 enrolled caregivers completed the full sequence of 6 calls. Field notes from each session were also reviewed to supplement the final narratives, providing context to each caregiver’s journey (for instance, noting improvements or setbacks observed by the nurse during the program).

### 2.5. Data Analysis

Quantitative analysis: Descriptive statistics were used to summarize the quantitative data. We calculated the mean and modal ages of both patients and caregivers, and the average duration and intensity of caregiving. For the SCB at baseline, the mean score was computed; according to the instrument’s guidelines, this was interpreted to categorize the overall burden level. Because of the small sample size and the primarily qualitative aims of the study, no inferential statistical tests were performed on quantitative measures. The six caregivers’ baseline mean SCB was 44 (indicating substantial burden). By the end of the program, the mean SCB had dropped to 36. No inferential statistical tests were performed due to the small sample and qualitative emphasis. Instead, the change in mean burden score (44 → 36) was examined descriptively (by comparing pre- and post-means) to assess potential impact. In other words, we interpreted the direction and magnitude of change rather than testing significance (consistent with outcome-focused pilot evaluation).

Qualitative analysis: All narrative feedback from the final sessions was subjected to a thematic analysis to identify recurrent themes in the caregivers’ experiences [17]. We followed the six-phase approach outlined by Braun and Clarke [17] for reflexive thematic analysis, which involved: (1) familiarization with the data, (2) generation of initial codes, (3) searching for themes among codes, (4) reviewing and refining themes, (5) defining and naming the final themes, and (6) producing the report. First, the caregivers’ narrative responses were transcribed verbatim from notes/recordings and read multiple times by the research team to ensure immersion in the content. Next, initial coding was carried out: meaningful units of text (phrases or sentences) were labeled with codes capturing their essence (for example, codes like “feeling of impotence,” “need for support,” “loss of patience,” “guilt feelings,” “recognizing personal strength,” “hope for future” emerged from different caregivers’ statements). The coding was primarily data-driven (inductive), given that we did not impose a predetermined framework but rather let the themes arise from the caregivers’ own words. The coded data segments were then collated and examined for patterns. Codes that related to each other were grouped into potential themes. For instance, codes concerning caregiver overwhelm, guilt, and exhaustion clustered into a theme regarding the emotional burden of caregiving; codes about needing support and seeking help formed a theme around recognizing external support needs; codes about patience, emotional regulation techniques, and communication issues formed a theme about managing the caregiver-patient interaction; and codes highlighting personal strengths and resilience coalesced into a theme on caregiver empowerment. These candidate themes were reviewed against the raw data to ensure they accurately reflected the caregivers’ narratives and that no significant ideas were overlooked. Through iterative discussion and refinement, the themes were clarified and named in a way that conveyed the core message of each. Ultimately, a set of key themes emerged that encapsulated the caregivers’ perspectives on how the program affected them.

The lead researcher maintained a reflexive journal throughout data collection and analysis. To enhance the rigor of the analysis, the coding and theme development were reviewed by a second experienced qualitative researcher who was not involved in the intervention delivery. Any discrepancies in interpretation were discussed until consensus was reached, thereby strengthening the credibility of the findings. NVivo 12 software (QSR International, Melbourne, Australia) was used to assist in organizing the qualitative data and managing codes, although coding was performed manually by reading transcripts. Thematic analysis was chosen for its flexibility and suitability in extracting insights from personal narratives, and it allowed us to integrate both anticipated outcomes and unexpected insights from the caregivers’ stories. Saturation was considered reached after six participants, given the homogeneity of the sample (informal caregivers of inpatients with bipolar disorder), the focused nature of the intervention, and the recurrence of themes across narratives. Direct quotes are presented to illustrate each theme, with caregivers identified anonymously as IC1 through IC6.

### 2.6. Ethical Considerations

This study was conducted in accordance with ethical standards for research involving human participants. The project was reviewed and approved by the institutional ethics committee of HDS (June 2021) prior to implementation. All participating caregivers provided informed consent for their involvement in the support program and for the use of their de-identified feedback in the evaluation. Participation in the program was voluntary, and caregivers were informed that they could withdraw at any time or decline to answer any questions during the calls without any impact on the care their relative received. Confidentiality of the data was strictly maintained: caregivers’ identities and personal details were kept confidential in all records, and findings are reported in aggregate or with non-identifiable quotes. The nurses conducting the telephone sessions were trained in privacy and ethical handling of sensitive information. Given the vulnerable context (caregivers of psychiatric inpatients), additional care was taken to ensure that the intervention did not pose psychological risks to participants; in fact, the sessions were designed to be supportive and to potentially reduce stress. After the conclusion of the study, the caregivers were provided with information on how to continue seeking support (such as contact information for family support groups and mental health services). The outcome of this program evaluation has been shared with the hospital administration and staff, and the intention is to integrate the telephone support as a regular offering for caregiver support in the psychiatric unit.

## 3. Results

Between June and August, a total of 34 patients were admitted to the HDS psychiatric inpatient services, 6 with BD. The patients’ mean age was 48 years (mode 53 years). The most frequent nursing diagnoses on admission were Impaired Communication, Impaired Mood, and Impaired Thought Processes. The quantitative data about the participating caregivers is summarized in Table 1.

The participating caregivers were predominantly female (4) with a mean age of 51 years. On average, they had been in a caregiving role for approximately 5 years and devoted around 5 h per day to caregiving tasks. This indicates a considerable caregiving commitment in the sample.

A total of 36 intervention sessions were delivered as planned. This included 6 initial assessment sessions, 18 psychoeducational sessions, 6 psychotherapeutic sessions, and 6 final evaluation sessions.

At baseline, the SCB mean score was 44, which falls within the range indicative of elevated caregiver burden; by the end of the program, it had fallen to 36. Given the small sample, this reduction was interpreted descriptively rather than statistically. The eight-point drop suggests a potentially meaningful improvement in perceived burden, consistent with caregivers’ qualitative reports of better coping.

The qualitative data from caregiver narratives were analyzed using thematic analysis [17]. This analysis yielded four recurrent themes that illustrate the caregivers’ experiences and perceived impact of the program:

Thematic analysis of the caregivers’ feedback yielded four recurrent themes, summarized in Table 1. These themes capture the caregivers’ experiences and perceived effects of the program: Embracing the Caregiver Role (Recognizing personal needs and legitimizing help-seeking); Looking at the Other with Patience and Understanding (Adopting emotion-regulation strategies to improve interactions with the patient); Self-Reflection on Personal Strengths (Increased self-efficacy and acknowledgement of persistence, empathy, and resilience); Fostering Hope and Resilience (Expressing future-oriented goals and maintaining optimism, consolidated in a personalized “hope kit”). The themes and representative quotations are detailed in Table 2.

### 3.1. Theme 1—Embracing the Caregiver Role

Caregivers described an emotional journey of acknowledging their role and the need for support. For example, one participant realized “I need a cane” (IC1)—a metaphor for requiring assistance—which symbolized the recognition of needing external help to continue caring effectively. Many caregivers voiced a heightened sense of responsibility toward their loved one: “I feel I must help and have to be more attentive” (IC1); “I keep thinking whether my help is enough” (IC4). Some linked this responsibility to lifelong family roles (“I’ve always felt this, as if I were her mother”—IC3). Caregivers also openly recognized their feelings of overload and impotence in the face of the illness: “I want to care for him, but I’m exhausted, and at the same time he has no one else” (IC1); “I feel powerless for not being able to make her get better” (IC4). Despite fatigue and frustration—“being at peace with the decision to care, but ending up exhausted and sad… feeling guilty for losing my patience” (IC2)—they remained committed: “I am the caregiver because I know he needs me” (IC2). This theme highlights the realization of needing support and resilience to uphold the caregiver role.

### 3.2. Theme 2—Looking at the Other with Patience and Understanding

Caregivers reported challenges in distinguishing their own emotions from feelings, yet understood that managing these emotions is crucial for maintaining a balanced relationship with the patient. They discussed how the stress of the caregiving situation—particularly struggles with the patient’s adherence to treatment—often tested their patience. For instance, one noted, “After discharge, he stops taking his medication and I lose my patience” (IC1), while another admitted, “I end up lashing out, and our body language shows it” (IC2). All participants found the introduction of specific coping techniques (such as the “turtle technique” of stepping back and the 10 s rule before reacting) to be useful strategies. These techniques helped them moderate their emotional responses, leading to improved patience and communication with their loved ones.

### 3.3. Theme 3—Self-Reflection on Personal Strengths

Through the program’s reflective exercises, caregivers identified and took pride in their personal strengths and positive qualities. They described traits such as foresight (“having the perspicacity to anticipate things”—IC1), honesty (“being truthful”—IC2), dedication (“being committed”—IC4), empathy and friendship (“being humane and a true friend”—IC3; “being a good friend”—IC1), resilience (“overcoming problems”—IC2), giving good advice (“being a good counselor”—IC1), persistence (“being persistent”—PC4), and readiness to help (“always being ready to help”—IC10). This process of acknowledging strengths was novel and empowering for many: “It’s so good to think about what we are good at, to recognize our capabilities” (IC2). Caregivers noted that each session’s conversation prompted new self-discoveries and personal growth, with one stating, “Every time we talk, I reflect and discover more things about myself” (IC2). They also examined their internal dialog and self-criticism, discussing ways to improve self-efficacy by cultivating optimism and humor. This theme reflects enhanced self-awareness and confidence among caregivers as a result of the program.

### 3.4. Theme 4—Fostering Hope and Resilience

In the final sessions, caregivers engaged in a creative visualization exercise—imagining a magic wand that could fulfill a wish—which elicited responses focused on hope, strength, and endurance. Caregivers “wished” for things like “hope” (IC1), “peace, love, and patience” (IC2), “not giving up” (IC3), “valuing life” (IC4), “having strength and courage” (IC3), “health and more capacity to help” (IC2), “time and ability to stay present and attentive” (IC3), “health, strength, and good humor” (IC1), “more understanding and tranquility” (IC1), and “courage and persistence” (IC4). This exercise created a shared vision of hope for the future and underscored the caregivers’ resilience. It also laid the groundwork for assembling a “hope kit,” a compilation of the group’s shared reflections and wishes (some of which participants wrote down during sessions). The magic wand metaphor, coupled with the hope kit, reinforced the idea that while external support is valuable, the strength to persevere also comes from within. Caregivers concluded that maintaining hope, courage, and a positive outlook are essential as they continue caring for their loved ones.

The individual reductions in SCB scores paralleled qualitative reports of greater emotional regulation, validation, and hope, suggesting coherence between subjective improvement and measurable burden change.

Taken together, the quantitative and qualitative data suggest that the nurse-led telephone psychoeducation program was associated with reduced subjective burden; improved emotion regulation, coping, and hope; and greater confidence in caregiving. These convergent findings provide preliminary evidence supporting the feasibility and potential effectiveness of remote, nurse-delivered support for families affected by BD.

## 4. Discussion

This pilot case study corroborates that family caregivers of individuals living with BD experience substantial burden. Recent investigations consistently document moderate-to-high levels of caregiver burden and, frequently, low resilience, and they recommend psychoeducational interventions to mitigate these pressures [18,19]. Narratives from our participants—exhaustion, powerlessness, and guilt (“I feel powerless for not being able to make her get better,” IC4)—mirror the well-characterized family impact of BD [18,19,20].

The telephone program proved both feasible and acceptable: all caregivers who initiated participation completed the six-call cycle. This high adherence suggests that flexible, accessible remote support addresses a genuine, unmet need. Comparable interventions report parallel benefits; for instance, a structured 12-session telephone psychoeducation program reduced caregiver burden and increased self-efficacy [21], and 16 scheduled telephone contacts were associated with improvements in caregivers’ depressive symptoms [22]. Although our study was qualitative in emphasis, these findings support the hypothesis that combining illness education with emotional containment can alleviate caregiver strain.

Evidence from comparable caregiver programs supports these observations. In dementia care, for instance, structured, scheduled telephone interventions have produced meaningful improvements in caregiver outcomes (e.g., reduced depressive symptoms and burden, increased self-efficacy), underscoring the potential of brief, protocolized calls to deliver both education and emotional containment. Although our evaluation was qualitative, these converging findings strengthen the plausibility that a nurse-led, telephone psychoeducation-plus-support model can mitigate caregiver strain. Moreover, recent syntheses across serious mental illness indicate that caregiver-focused psychoeducation and digitally supported or blended formats remain effective and acceptable, aligning with our participants’ reports of acquiring concrete coping strategies and greater confidence in the caregiving role [18,19,20,21,22].

Within BD specifically, the family-focused psychoeducational tradition continues to evolve, including technology-enhanced approaches that extend structured family work beyond the clinic (e.g., remote check-ins, digital tools to scaffold skills between sessions). Such innovations map closely onto the program implemented—combining illness education, emotion-regulation coaching, and problem-solving—while leveraging remote delivery to preserve continuity and reach. Feasibility and acceptability data from recent youth-focused tele-psychotherapy studies, and implementation work in health systems, further suggest that virtual contact can sustain therapeutic alliance and adherence when carefully structured—points that dovetail with the high completion we observed.

Moreover, reviews and meta-analyses in BD indicate that family psychoeducation reduces perceived burden and increases caregivers’ knowledge [23,24,25], aligning with participants’ reports of acquiring coping strategies and greater confidence in the caregiving role. More recent syntheses (2021–2024) further corroborate the effectiveness of caregiver-focused psychoeducation and blended tele-supports in serious mental illness [26,27,28,29].

The observed improvements can be conceptualized through key mechanisms of change embedded within the intervention. Emotional validation likely alleviated distress by legitimizing caregivers’ feelings of frustration and guilt, reducing emotional isolation, and promoting adaptive coping. Structured discussions around communication strategies may have enhanced caregivers’ interpersonal efficacy, facilitating clearer, less conflictual exchanges with their relatives. Normalization—framing their experiences as common among families affected by BD—appears to have reduced self-blame and fostered a sense of shared understanding. Collectively, these mechanisms align with Lazarus and Folkman’s stress–coping framework [30], wherein reappraisal, problem-focused coping, and emotion regulation mediate stress outcomes. Similarly, within Pearlin’s caregiver stress process model [30], the program can be viewed as targeting both primary stressors (illness-related demands) and secondary strains (role overload, emotional exhaustion) through skill-building and psychosocial support, thereby strengthening coping resources and perceived mastery.

At program completion, caregivers were invited to provide feedback on satisfaction and perceived usefulness. All participants reported that the telephone format was convenient and accessible, and four of five rated the experience as “very helpful.” Perceived benefits included increased understanding of bipolar disorder, emotional support, and feeling “less alone” in the caregiving process. No participants withdrew or expressed dissatisfaction, supporting the program’s acceptability.

It is important to acknowledge that the same nurse who conducted the intervention also led data collection and analysis. While this continuity may have enhanced rapport and depth of disclosure, it also introduces potential bias, as participants might have moderated their feedback or emphasized positive aspects. This dual role underscores the need for reflexive awareness in interpreting findings and highlights the value of future studies incorporating independent evaluators to strengthen methodological rigor.

Post-Program Integration and Sustainability

Following the completion of the program, several steps were taken to consolidate its success and integrate the intervention into routine practice. A collaborative debriefing session was conducted with the nursing team and the supervising nurse of the psychiatric unit to share the program’s outcomes and the caregivers’ feedback. This session aimed to recognize the successes achieved and to encourage the team’s engagement in continuing the intervention. Additionally, educational and procedural materials were developed to standardize the approach: a pamphlet was created to disseminate information about the program, a flowchart was designed to guide nurses through the intervention steps, and a formal protocol was written to incorporate the program into the unit’s care processes. It was also proposed that a specific nurse be designated as the program coordinator within the unit, responsible for overseeing ongoing implementation and providing support to colleagues. Through these measures—team training, resource development, and leadership designation—the program’s practices were standardized and sustained, ensuring that the benefits to caregivers can be maintained and that successful outcomes are recognized and built upon.

### 4.1. Limitations

This is a pilot case without a control group and with a small sample (6 informal caregivers). Larger, preferably randomized, studies are needed to quantify the benefits of telephone programs for BD caregivers. Nonetheless, our findings support investment in formal caregiver support and echo guidance that family psychoeducation is a core component of BD care. Nevertheless, the absence of a standardized satisfaction scale represents a limitation, and future trials should include quantitative evaluation of user experience and cost-effectiveness outcomes.

### 4.2. Implications for Nursing Practice

Structured telephone interventions reduce caregiver burden and are reinforced by recent telehealth trials. Train nurses in psychoeducation and telephone counseling, including screening for caregiver exhaustion and teaching emotion-regulation skills, in line with recommendations for family involvement. Therapeutic-plan integration. Involve caregivers in discharge planning, link them to supports (family groups, community services, helplines), and document needs. Continuous monitoring. Maintain follow-up and progress notes to adapt support and refer when needed; recognizing the caregiver as a care partner improves adherence and continuity.

Future controlled studies should quantify effect sizes on caregiver burden, depressive symptoms, and resilience, and test maintenance strategies (e.g., tapered booster calls, blended digital resources) to consolidate gains over time.

## 5. Conclusions

This pilot study demonstrates that a nurse-led telephone support program for informal caregivers of individuals with bipolar disorder is both feasible and acceptable. The program showed high adherence and meaningful qualitative benefits. Caregivers described reduced subjective burden, improved emotion regulation, recognition of personal strengths, and renewed hope—outcomes that align with previous evidence showing the effectiveness of psychoeducation and telephone-based interventions in mitigating caregiver strain and enhancing resilience. The findings corroborate reviews and meta-analyses indicating that family psychoeducation reduces perceived burden, increases caregiver knowledge, and improves coping, while more recent syntheses highlight the effectiveness of blended and telehealth-based formats in supporting caregivers of individuals with serious mental illness.

Telephone-based interventions offer a low-cost, scalable model that can improve access equity, particularly for caregivers facing geographic or financial barriers. For nursing practice, these results underscore the role of mental health nurses as key providers of psychoeducation, emotional support, and empowerment strategies for families, with potential scalability across diverse care contexts. Structured remote interventions also offer advantages in cost-effectiveness and access equity, extending specialized support to caregivers who face geographic, financial, or time-related barriers to in-person care. Although limited by the small sample size and absence of a control group, this study contributes practice-based evidence from a Portuguese psychiatric inpatient setting, demonstrating that structured telephone support can bridge gaps in traditional caregiver assistance. Future research should include larger randomized controlled trials to quantify impacts on caregiver burden, depressive symptoms, and resilience, and to examine sustainability and optimal follow-up strategies—such as booster calls or integration with digital resources. By recognizing caregivers as essential partners in care and equipping them with knowledge, coping strategies, and emotional validation, nurse-led telephone programs can foster more resilient families and enhance continuity of care for individuals living with bipolar disorder.

## Figures and Tables

**Table 1:** Caregivers’ quantitative data.

Caregiver	Gender	Age	Years in Caregiving Role	Daily Care Hours	SCB Score (Pre)	SCB Score (Post)
IC1	Female	53	5	7	45	37
IC2	Female	42	6	2	43	36
IC3	Male	58	3	6	44	35
IC4	Female	50	4	4	46	38
IC5	Male	54	5	8	42	34
IC6	Female	48	6	2	44	36

**Table 2:** The themes and representative quotations.

Theme	Representative Quote(s)
1. Embracing the caregiver role	“I feel I must help and have to be more attentive” (IC1)
2. Looking at the Other with Patience and Understanding	“I end up lashing out, and our body language shows it” (IC2)
3. Self-reflection on Personal Strengths	“It’s so good to think about what we are good at, to recognize our capabilities” (IC2).
4. Fostering hope and resilience	“having strength and courage” (IC3), “health and more capacity to help” (IC2), “time and ability to stay present and attentive” (IC3)

## Data Availability

The original contributions presented in this study are included in the article. Further inquiries can be directed to the corresponding author.
